# Audio-Visual Speech Timing Sensitivity Is Enhanced in Cluttered Conditions

**DOI:** 10.1371/journal.pone.0018309

**Published:** 2011-04-06

**Authors:** Warrick Roseboom, Shin'ya Nishida, Waka Fujisaki, Derek H. Arnold

**Affiliations:** 1 School of Psychology, The University of Queensland, Brisbane, Australia; 2 NTT Communication Science Laboratories, Nippon Telegraph and Telephone Corporation, Atsugi-shi, Kanagawa, Japan; 3 Human Technology Research Institute, National Institute of Advanced Industrial Science and Technology (AIST), Higashi, Tsukuba, Ibaraki, Japan; University of Sydney, Australia

## Abstract

Events encoded in separate sensory modalities, such as audition and vision, can seem to be synchronous across a relatively broad range of physical timing differences. This may suggest that the precision of audio-visual timing judgments is inherently poor. Here we show that this is not necessarily true. We contrast timing sensitivity for isolated streams of audio and visual speech, and for streams of audio and visual speech accompanied by additional, temporally offset, visual speech streams. We find that the precision with which synchronous streams of audio and visual speech are identified is *enhanced* by the presence of additional streams of asynchronous visual speech. Our data suggest that timing perception is shaped by *selective* grouping processes, which can result in enhanced precision in temporally cluttered environments. The imprecision suggested by previous studies might therefore be a consequence of examining isolated pairs of audio and visual events. We argue that when an isolated pair of cross-modal events is presented, they tend to group perceptually and to seem synchronous as a consequence. We have revealed greater precision by providing multiple visual signals, possibly allowing a single auditory speech stream to group *selectively* with the most synchronous visual candidate. The grouping processes we have identified might be important in daily life, such as when we attempt to follow a conversation in a crowded room.

## Introduction

Determining the simultaneity of events occurring in multiple sensory modalities is a conceptually challenging task. The environment in which humans exist is cluttered, with many events occurring in close spatial and temporal proximity. This situation is exacerbated by differences in transmission times for auditory and visual information that originate from a single physical event. These can arise due to both physical transmission speed differences and because of signal intensity related variations in propagation speeds through the central nervous system (see [1]–[3]).

One way to deal with such variability when judging simultaneity would be to adopt a broad criterion, with auditory and visual signals judged as synchronous across an extended range of physical timing differences. Psychophysical evidence is broadly consistent with the brain having adopted such a strategy, with audio and visual events being judged as synchronous when separated by up to ∼200 ms [4]–[9]. The apparent simultaneity of auditory and visual events is further encouraged by temporal ventriloquism, in which the apparent timing of cross-modal events is drawn *toward* one another [10]–[12]. Interestingly, the range of physical timing differences across which audio and visual events can seem synchronous is shaped by content, with audiovisual (AV) speech likely to be judged as synchronous across a broader range of timing differences than more basic stimuli, such as light flashes and beeps [2], [4], [13]. This has been linked to the ‘unity assumption’, wherein the apparent relatedness of two events can shape the range of timing differences across which they are likely to be judged as synchronous [4], [8]–[9], [13]–[16]. Changes in this range can also occur due to learning and previous experience [17]–[22].

The expanse of timing offsets across which audio and visual signals seem synchronous is often referred to as the AV simultaneity window [2], [23]–[24]. The implication of this terminology is that visual and audio signals will only be reliably judged as *asynchronous* if they are separated by a period greater than the extent of the AV simultaneity window. The width of the AV simultaneity window is often taken as a measure of temporal resolution, with broad windows taken as evidence for a coarse resolution.

Recently it was suggested that the coarse resolution suggested by many AV timing experiments might, to some degree, be a consequence of experimental design [25]. Specifically, it was pointed out that almost all AV timing experiments examine isolated pairs of audio and visual events, whereas in daily life we encounter scenarios in which many audio and visual events occur in close temporal proximity. There may be a tendency for isolated pairs of events to group, thereby enhancing perceived synchrony, whereas perceptual grouping and timing perception might be more selective in cluttered conditions. Accordingly, the true resolution of human AV timing perception might only become apparent via experimental designs that incorporate multiple possible AV pairings [25].

Speech is perhaps the most important AV signal for humans. It is also subject to some powerful cross-modal interactions. These seem to occur at an early neural locus, as visual speech can impact neural latencies for corresponding auditory information [28], [29]. AV speech interactions can also shape what is heard (e.g. McGurk-MacDonald effect; [26]), and improve speech comprehension via sensory integration [27]. Importantly in this context, the spatial and temporal origins of audio and visual speech tend to be drawn toward one another – phenomena known respectively as spatial [30]–[31] and temporal ventriloquism [10]–[12]. Such operations almost undoubtedly contribute to isolated AV speech cues being judged as synchronous across large timing differences. Here we are interested in whether this indicates that humans are simply insensitive to AV speech *asynchrony*, or if previous experiments might have underestimated human AV timing acuity by examining only isolated AV speech cues.

To examine this issue we contrasted AV timing sensitivity for auditory and visual speech signals presented in isolation, or for auditory and visual signals presented in the company of an additional, asynchronous, visual speech stream.

## Methods

### General Methods

#### Ethics Statement

Participants consisted of members of the University of Queensland Perception Lab. Before the experiment, participants were provided with an information sheet which outlined the general purpose of the study and informed them that they could withdraw at any time without penalty. As all participants were associates of the Lab, they were generally familiar with the basic methods employed in these experiments. As such, participants were only required to give verbal consent before the experiment began. All methods employed in this study were approved by The University of Queensland School of Psychology Ethics committee, and were in accordance with the Declaration of Helsinki.

#### Participants

In Experiment 1 participants included two of the authors and an additional five volunteers. In Experiment 2 participants included two of the authors and an additional four volunteers. In Experiment 3 participants included one of the authors and an additional eight volunteers. In Experiment 4 participants included one of the authors and an additional six volunteers. All volunteers were naïve as to the experimental purpose and all participants reported having normal hearing and normal, or corrected to normal, vision.

#### Apparatus

All experiments were run on a Dell Pentium 4 PC. Visual stimuli were displayed on a gamma-corrected 21″ Samsung SyncMaster 1100p+ monitor (resolution of 1024×768 pixels and refresh rate of 120 Hz) and were generated using a ViSaGe from Cambridge Research Systems. Participants viewed stimuli from a distance of 57 cm, with their head placed in a chinrest. Audio signals were presented diotically via Sennheiser HDA 200 headphones and were generated using a TDT Basic Psychoacoustic Workstation (Tucker-Davis Technologies). Audio presentations were synchronised with the visual display using triggers from the ViSaGe, timed to coincide with a monitor refresh. Participants' responses were recorded using a CRS CB6 Response Box.

## Results

### Experiment 1

In Experiment 1, we contrasted performance in two different two-alternative-forced-choice (2AFC) tasks. The tasks involved presentations of two visual events (video footage of an actor saying ‘ba’), and either one or two audio events (the sound of the same actor saying ‘ba’). This enunciation was chosen as it constitutes a voiced bilabial stop, so the dynamics of the visual lip movements are relatively clear.

The critical manipulation was whether the two visual event streams were presented concurrently (in two separate spatial locations – a spatial 2AFC task; see Movie S2) or sequentially (in discrete intervals separated by 1000 ms – a temporal 2AFC task; see Movie S1).

#### Methods

**Figure 1:**
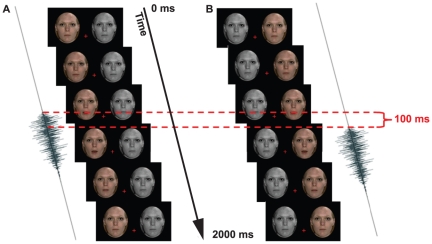
Graphical depiction of one possible Sequential stimulus presentation from Experiment 1. Only one face was animated at a time. To accentuate this, here the unanimated face is shown in grey-scale, for illustration purposes. Audio and visual streams were synchronous in one presentation (A). In the other presentation, the auditory stream trailed the visual stream by some offset (B).

The basic stimulus consisted of a two second AV recording of a female saying /ba/ (recorded using a Sony HDRSR12 Handycam). The visual portion of the recording was duplicated, with each visual stream centered 1.1 degrees of visual angle (dva) to either side (see Figure 1 or Movies S1 & S2) of a central crosshair fixation point (subtending 0.4 dva). Timing offsets between the two animated streams were introduced by repeatedly presenting the initial animation frame in just one of the visual streams. These individual frames were then presented in sequence at 60 frames per second, using a ViSaGe, stimulus generator from Cambridge Research Systems. Each visual stream was viewed through a 3.7 dva wide and 4.2 dva high oval aperture. The background was black (∼0 cd/m^2^). Audio signals were produced from the original audio recording (16 bit sample size, mono), which was normalized to a peak sound intensity of ∼65 db SPL with a “Hiss and Hum” filter applied which removed any section with a peak not exceeding 20 db (using WavePad Audio Editor, NCH Software).

In Sequential (temporal 2AFC) trials, only one of the two visual streams was animated at a time, while a static image was presented in the other location (see Figure 1A–B and Movie S1). The visual and auditory streams were physically synchronous in only one of the two sequential presentations. In the other, the auditory stream was physically offset, either leading or lagging the visual stream. The order of presentation, synchronous first or second, was randomized on a trial-by-trial basis. The side of visual animation, right or left, was randomized on a presentation-by-presentation basis. Participants were required to report, via a button press, in which of the two sequential presentations the audio and visual streams had been synchronous – a temporal 2AFC task.

**Figure 2:**
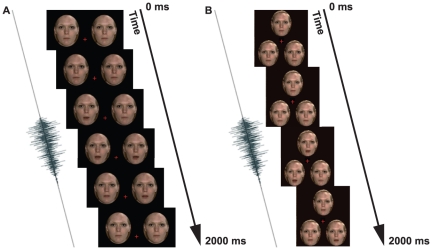
Graphical depictions of stimuli from Experiments 1 and 2. (A) Concurrent presentation from Experiment 1. Two temporally offset visual streams were presented. The auditory stream was synchronous with one of the two visual streams. (B) Concurrent presentation from Experiment 4. Three temporally offset visual streams were presented. The auditory stream was synchronous with one of the three visual streams.

In Concurrent (spatial 2AFC) trials two visual streams were presented, one on either side of fixation, along with a single audio stream (see Figure 2A; Movie S2). The audio stream was synchronized with one of the two visual streams – and was either leading or lagging the other visual stream. The position of the synchronized visual stream, left or right of fixation, was randomized on a trial-by-trial basis. Participants were required to report which of the two visual streams, left or right, the audio stream had been synchronous with – a spatial 2AFC task.

In all trials, each presentation started with static images on both sides of fixation. This persisted for 1000 ms +/− a pause of up to 500 ms, determined on a trial-by-trial basis. The inclusion of this variable initial pause avoided participants judging AV timing relationships on the basis of the delay between the start of the trial and the presentation of the audio event. Stimulus presentations persisted for 2 seconds after the initial variable pause. Participants were required to wait until the completion of the trial animation(s) before making their response. Feedback as to the accuracy of response was provided after each trial to maintain participant motivation and maximize performance.

One of the authors (WR) and another participant (AD), who was naïve as to the experimental purpose, completed the experiment with asynchronous AV stimuli separated across a range of offsets (+/−33, 66, 100, 133, 166, 200, 233 ms; AD completed additional blocks of trials at +/−300 ms). For each temporal offset, two blocks of 60 trials were completed for the Sequential condition, and a single block of 120 trials for the Concurrent condition. Blocks of trials were completed in a Sequential-Concurrent-Sequential order to minimize practice effects. Five further participants each completed two blocks of 60 trials for the Sequential condition, and a single block of 120 trials for the Concurrent condition with asynchronous AV stimuli separated by +/−100 ms.

#### Results

**Figure 3:**
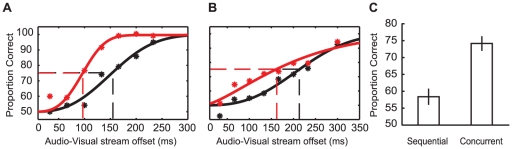
Results from Experiment 1. (A) Weibull functions fit to the results of author WR testing at a range of audio-visual offsets. Black data points are from Sequential presentations, while red data points are from Concurrent presentations. Broken lines show the audio-visual offset required to reach the 75% threshold for Sequential (black) and Concurrent (red) presentations. (B) As for (A) though for participant AD. (C) Bar plot depicting proportion of correct AV synchrony judgments. Data are shown for Sequential and Concurrent presentations in two-alternative forced choice tasks (Experiment 1). Error bars show standard error of the mean.

The proportion of correct responses, for both the Sequential and Concurrent conditions, were plotted as a function of asynchronous AV offsets and fit with Weibull functions (using psignifit toolbox version 2.5.6 for Matlab; see [32]). As shown in Figure 3A, for WR the AV timing offset required to differentiate AV synchrony from asynchrony on 75% of trials was approximately 96 ms in the Concurrent condition and 153 ms in the Sequential, a difference of 57 ms. Data for AD showed a similar pattern (Concurrent threshold = 162 ms; Sequential threshold = 213 ms; 51 ms difference; Figure 3B). On the basis of these data, a second author (DA) and a further four participants, naïve as to the experimental purpose, were tested at a single AV timing offset (+/−100 ms). As shown in Figure 3C, these participants correctly identified the synchronous AV pairing more often in the Concurrent condition (74% +/−4) than in the Sequential (58% +/−4; paired samples *t_6_* = 6.04, *p*<0.001). This shows that AV speech timing sensitivity can be *enhanced* by the presence of additional, temporally proximate, sensory events.

### Experiment 2

The results of Experiment 1 demonstrate that the presence of an additional visual speech stream can improve AV speech timing sensitivity. However, given that the window of AV simultaneity is shaped by stimulus type [2], [4], [13] and exposure [17], [18], [19], [20], [21], [22], it is possible that the processes underlying this improvement might not generalise to all AV pairings, but may be limited to highly learned stimuli, such as AV speech. We therefore repeated the experiment using more basic AV signals; brief visual presentations of luminance modulated Gaussian blobs and auditory presentations of frequency modulated tonal pips. Depending on the task, participants made judgments as to whether a single audio event had been synchronous with one of two concurrent visual events (Concurrent condition – a spatial 2AFC), or if the first or second AV presentation had been synchronous (Sequential condition – a temporal 2AFC).

#### Methods

**Figure 4:**
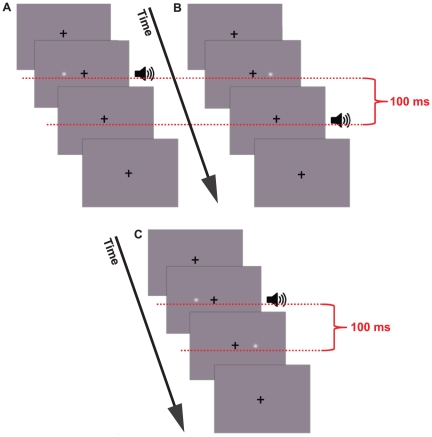
Graphical depictions of the stimuli from Experiment 2. (A–B) Depiction of one possible Sequential stimulus presentation. Only a single Gaussian blob was present on a single presentation. Audio and visual events were synchronous in one of the presentations (A). In the other presentation, the auditory event trailed the visual event by 100 ms (B). (C) Depiction of the Concurrent Condition. Two visual events were present offset by 100 ms. The single auditory event was synchronous with just one visual event.

Visual events consisted of a luminance modulated Gaussian blob (0.33 dva in diameter; peak luminance difference from background was 49 cd/m^2^, Michelson Contrast = 0.31; see Figure 4) displayed against a grey (54 cd/m^2^) background. A black (∼0 cd/m^2^) crosshair (subtending 0.4 dva) was presented centrally with the blob appearing 1.5 dva to the right or left of the crosshair. The blob was presented for 8.33 ms following a pseudo-random delay of up to 725 ms from the beginning of the presentation. Throughout each trial a 400 Hz tone (∼68 dB SPL) was presented. Auditory events consisted of an 8.33 ms pulse of a 900 Hz tone (a transitory change from 400 to 900 Hz). The duration of each individual presentation was 833 ms. Authors WR and DA along with a further four participants, naïve as to the experimental purpose, were tested at a single AV timing offset (+/−100 ms). The remaining Methods were identical to that of Experiment 1.

#### Results

Participants correctly identified the synchronous AV presentation on 85% (+/−3%) of Concurrent trials and on 78% (+/−5%) of Sequential trials, revealing a 7% advantage for Concurrent stimulus presentations (*t_5_* = 4.55, *p* = 0.006, paired samples – two tailed). This indicates that the results of Experiment 1, revealing an advantage for AV timing sensitivity in the presence of additional, temporally proximate, sensory events, is not limited to stimuli with complex temporal profiles, such as AV speech, but can also be found when using very basic stimuli.

### Experiment 3

One possible criticism of the results of Experiments 1 and 2 is that the difference in performance between Sequential and Concurrent conditions may be attributable to differences in memory load between the two conditions. The two intervals in the Sequential condition may require that participants keep in memory an estimate of the AV timing relationship in the first interval in order to compare it with the second interval. The Concurrent condition does not contain the same memory requirement. To address this issue we conducted a further experiment using a signal detection methodology similar to that employed in [25].

#### Methods

The methods of Experiment 3 were the same as those for Experiment 1 with the following exceptions. In Experiment 3 there were two conditions: Baseline and Concurrent. In Baseline trials a single visual speech stream was presented, to the left or right of fixation. The corresponding auditory stream was presented synchronous with, preceding, or succeeding the visual stream. After viewing animations participants reported if visual and audio streams had been synchronous. The Concurrent condition was identical to that in Experiment 1.

One of the authors (WR) and another participant (RS), who was naïve as to the experimental purpose, completed the experiment with asynchronous AV stimuli separated across a range of offsets (+/−66, 100, 133, 166, 200, 233 ms). Participants completed two blocks of 120 trials for each temporal offset sampled. Each block of trials contained 60 Baseline and 60 Concurrent trials, pseudo-randomly interspersed. Seven further participants, all naïve as to the purpose of the study, completed two blocks of trials with asynchronous AV stimuli separated by +/−166 ms.

#### Results and Discussion

**Figure 5:**
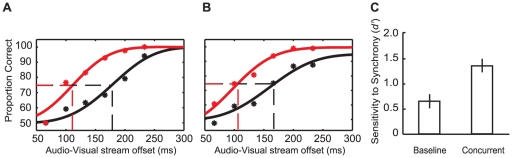
Results from Experiment 3. (A) Weibull functions fit to the results of author WR testing at a range of audio-visual offsets. Black data points are from Baseline presentations, while red data points are from Concurrent presentations. Broken lines show the audio-visual offset required to reach the 75% threshold for Baseline (black) and Concurrent (red) presentations. (B) As above, though for participant RS. (C) Bar plot depicting participants' sensitivity for detecting synchrony for Baseline and Concurrent conditions. Error bars show standard error of the mean.

For WR and RS, the proportion of correct responses for Baseline and Concurrent trials were plotted as a function of asynchronous AV offsets and fit with Weibull functions. As can be seen in Figure 5A, the AV timing offset required for WR to determine which AV pair had been synchronous on 75% of trials was approximately 113 ms in the Concurrent condition. To decide if the AV presentation had been synchronous or asynchronous in the Baseline condition required an AV timing offset of 178 ms, a difference of 64 ms. RS showed a similar pattern of results, with discrimination thresholds of 103 ms in the Concurrent condition and 161 ms in the Baseline condition, a difference of 58 ms (see Figure 5B). As these data only compare the proportion of correct responses in each condition, they could have reflected a more conservative response criterion in the Baseline condition, leading to an apparently increased threshold rather than a genuine difference in sensitivity. However, for this to be true the false alarm rate for Baseline responses would have to approach 0%. This was not true of the data for RS or WR, for which the average false alarm rates (across all AV offsets) were 45% and 39% respectively, with minima of 20% (for RS at 200 ms AV offset) and 10% (for WR at 233 ms AV offset). Therefore it is unlikely that a conservative response criterion in the Baseline condition could account for the observed difference in sensitivity.

Individual data from an additional seven participants, tested at a single AV timing offset (+/−166 ms), were converted into measures of hit rate (HR), false alarm rate (FAR) and sensitivity (*d′*) as per signal detection theory (SDT; [25], [33], [34]). Baseline data were converted to *d′* according to *d′* = [*z*(HR)−*z*(FAR)] where HR is the proportion of ‘synchronous’ reports when synchrony was presented and FAR is the proportion of ‘synchronous’ reports when the presentation was asynchronous. Concurrent data were converted to *d′* according to *d′* = [*z*(HR)−*z*(FAR)]/√2 where HR is the proportion of ‘synchronous left’ reports when the visual speech stream on the left of fixation was presented in synchrony with the auditory stream and FAR is the proportion of ‘synchronous left’ reports when the visual speech stream on the right of fixation was presented in synchrony with the auditory stream. As shown in Figure 5C, participants' AV timing sensitivity was significantly greater in Concurrent (*d′* = 1.34; *s.e.m.* = 0.25) than in Baseline trials (*d′* = 0.64; *s.e.m.* = 0.26; paired samples *t_8_* = 4.83, *p* = 0.002). As such, participants were superior at deciding which of two visual speech streams had coincided with the auditory stream than they were at deciding whether or not isolated visual and audio streams had been coincident.

As is true of all studies employing a standard SDT model, our sensitivity estimates (*d′*) are dependent on several key assumptions. First, it is assumed that participants' response criteria remain fixed throughout testing, something we tried to encourage through the provision of trial-by-trial feedback. However, as with all protracted experiments, it is possible that this assumption was violated. Second, it is assumed that the variance associated with sensory signal and noise distributions is equal. Again, it is possible that this assumption was violated. However, despite these caveats, we believe that the consistency of results across different experimental designs points to a robust effect, wherein AV timing sensitivity is enhanced in the presence of an additional asynchronous visual signal. As such, it seems unlikely that this effect reflects a difference in memory load, particular to the design of Experiments 1 and 2, and/or a violation of the assumptions underlying standard models of SDT, particular to Experiment 3.

### Experiment 4

The results of Experiments 1 to 3 showed that AV timing sensitivity could be *enhanced* by the presence of an additional, temporally proximate, visual event. In Experiment 4 we decided to investigate if the same results could be obtained in the presence of even greater temporal clutter, expanding the task from Experiment 1 to encompass three visual event streams.

#### Methods

The methods of Experiment 4 were as for Experiment 1, with the following exceptions. Three streams of visual animation were used. The possible locations of the visual streams were arranged in a triangular formation (see Figure 2B), such that two visual streams were on the bottom, centered 1.5 dva either side of and 1.2 dva below a central fixation cross hair (which subtended 0.4 dva). A third stream was centered horizontally, 2.0 dva above the cross hair. Each animation location was viewed through a 2.6 dva wide and 3.2 dva high oval aperture.

There were two conditions, Sequential and Concurrent. In Concurrent trials (a spatial 3AFC), three visual streams were presented concurrently, along with a single audio stream (see Figure 2B). The onset of movement in the three visual streams was offset, such that one (top, left or right – determined at random on a trial-by-trial basis) would begin to move first followed by another after a delay and then a third after an *additional* delay. The auditory stream was synchronized with just one of the three visual streams. The location of the synchronized visual stream (top, left or right) and the order in which the synchronized visual stream began to move (first, second or third) was determined at random on a trial-by-trial basis. Participants were required to report which of the three visual streams (top, left, or right) had been synchronized with the audio stream.

In Sequential trials (a temporal 3AFC), only a single animated visual stream was presented at a time, while alternate locations contained static images. In every trial one presentation contained a synchronous AV relationship, while the other two presentations contained asynchronous relationships. Asynchronous presentations could consist of: one presentation with an auditory *lead* and another with an auditory *lag*; one presentation with an auditory lead and another with a double sized auditory lead; or one presentation with an auditory lag and another with a double sized auditory lag. In this way, the possible audio - visual presentation offsets for Sequential trials were matched with those for Concurrent trials.

Each presentation was separated by a 1000 ms ISI, with the order of presentation (i.e. whether the synchronous pair was presented first, second or third in the trial sequence) determined at random on trial-by-trial basis. The location of the visual streams always progressed clockwise from the left position. Participants were required to identify in which presentation, first, second or third, the AV signal streams had been synchronized.

One of the authors (WR) and another participant (AD), who was naïve as to the experimental purpose, completed runs of trials with delays between successive visual stream onsets set to 33, 100, 133, 166, 200 or to 233 ms. A further five participants, also naïve as to the experimental purpose, completed trial runs with delays between successive visual stream onsets of 166 m. Given the low level of chance performance in this experiment (33% as opposed to 50% in Experiment 1), we increased the offset between asynchronous AV speech streams (to 166 ms as opposed to 100 ms in Experiment 1). This was designed to maintain the motivation of our participants.

For each offset, naïve participants completed three blocks of 72 trials for the Sequential condition, and two blocks of 108 trials for the Concurrent condition. WR completed three blocks of 108 trials for the Sequential condition, and two blocks of 162 trials for the Concurrent condition.

#### Results

**Figure 6:**
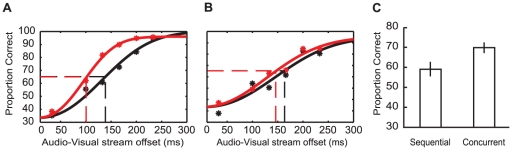
Results from Experiment 4. (A) Weibull functions fit to the results of author WR testing at a range of audio-visual offsets. Black data points are from Sequential presentations, while red data points are from Concurrent presentations. Broken lines show audio-visual offset required to reach the 66% threshold for Sequential (black) and Concurrent (red) presentations. (B) As for (A) though for participant AD. (C) Bar plot depicting proportion of correct AV synchrony judgments. Data are shown for Sequential and Concurrent presentations in three-alternative forced choice tasks (Experiment 4). Error bars show standard error of the mean.

For WR and AD proportions of correct responses were plotted as a function of AV offset, for both the Sequential and Concurrent conditions. Weibull functions were fit to the resulting distributions. As shown in Figure 6A, for WR the AV timing offset required to reach a 66% correct threshold (the mid-point between chance and perfect performance) was 101 ms in the Concurrent condition and 143 ms in the Sequential condition, reflecting an advantage of 42 ms for the Concurrent condition. AD (Figure 6B) showed a similar pattern of results (Concurrent threshold 144 ms; Sequential threshold 160 ms; 16 ms difference).

As shown in Figure 6C, the additional participants correctly identified the synchronous AV pairing in the Concurrent condition on 70% (+/−5) of trials, and on 59% (+/−3) in the Sequential condition, reflecting an advantage of 11% for Concurrent trials (paired samples *t_6_* = 4.24, *p* = 0.005).

## Discussion

Our data show that the precision by which synchronous streams of AV speech are identified can be *enhanced* by the presence of additional streams of asynchronous visual speech. This was shown by contrasting sensitivity during concurrent and sequential presentations of two (Experiment 1) or three (Experiment 4) visual speech streams. Additionally, we found that this enhancement in sensitivity was not limited to complex stimuli like AV speech (Experiments 1, 3 & 4), but could also be found using a basic stimulus (Experiment 2). Moreover, these results could not be attributed to differences in memory load between presentation conditions (Experiment 3). In all cases, AV timing sensitivity was enhanced in the presence of additional, temporally proximate, visual signals. This demonstrates the existence of interactions, involving concurrently presented visual signals, which can enhance AV timing sensitivity.

We have shown that the precision of AV timing judgments, at least for brief audio and visual events, can be greater than one would predict on the basis of judgments concerning isolated AV events. These observations may be somewhat counterintuitive, with an improvement in AV timing sensitivity resulting from an increased number of visual events (see [35]). We believe our results can be explained, at least in part, by a tendency for cross modal signals to group on the basis of temporal proximity. When an AV pair is presented in isolation, there may be a strong perceptual tendency to group these two events due to a lack of any other, temporally more proximate, candidate events. This scenario is well characterized by the ‘unity assumption’ [4], [8]–[9], [13]–[16]. When there are multiple, clearly asynchronous, intra-modal events (in this case different streams of visual speech), a cross-modal signal might group with the most temporally proximate candidate intra-modal event, bringing about a perceptual segregation of the now grouped events from other ungrouped signals. Such a process could be conceptualized as a *selective* form of temporal ventriloquism [10]–[12].

Our proposal is broadly consistent with the observation that when multiple auditory events group perceptually, to form a single stream of events (such as a sequence of tones), they have less impact on the apparent timing of visual events [36]–[39]. In both cases selective perceptual grouping, be it intra-modal (as in the studies mentioned above) or cross-modal (as in this study), seems to mitigate alternative cross-modal interactions involving the grouped events. Importantly, all events in this study, both auditory and visual, were clearly distinguishable as distinct signals, mitigating any possible intra-modal grouping effects.

An alternative proposal might be that the distribution of attentional resources, when multiple plausible AV pairings are possible, reduces attention to a given stimulus, thus diminishing the ‘unity assumption’ for a given AV pair. Such a process would be consistent with previous results demonstrating decreases in just noticeable differences when the perception of unity between an AV pair is diminished [9] and decreases in AV integration under conditions of high attentional demand [40]. Note, however, for such a process to result in the enhanced AV timing sensitivities we have discovered, the disruption of the unity assumption would have to be *selective* on the basis of temporal proximity.

An important feature of the experiments in this study was that the AV timing offsets fell within previous estimates of the AV simultaneity window for speech (up to ∼200 ms from physical synchrony, see [4]–[5]). These data are therefore consistent with the suggestion that the extent of the AV simultaneity window is dynamic, shaped by the presence of additional temporally proximate sensory events [25]. As such, our data are *inconsistent* with the premise that the AV simultaneity window reflects a fixed interval during which the detection of AV *asynchrony* is impossible. Our data demonstrate a clear improvement in AV timing sensitivity (see Figures 3, 5, 6) when a target visual signal is presented in conjunction with other asynchronous visual signals. This implies that not only is the window of subjective AV simultaneity dynamic [25], but that objective measures of AV timing sensitivity can also be impacted by the number of possible sensory matches. It remains to be seen if these objective changes in AV timing sensitivity will be reflected in the extent of sensory integration for a given AV pairing.

The improvement in AV timing sensitivity that we have identified may tap a similar process to that underlying another AV phenomenon – the ‘Pip and Pop’ effect [41]–[43]. In this effect, there is a small facilitation for the speeded detection of a visual target, embedded within a field of asynchronously changing visual distracters, when the target changes are coupled with transient tonal pips [41]. Similarly, our data show enhanced sensitivity to AV synchrony in the presence of concurrent, asynchronous, visual events. It could be argued that, in both cases, synchronous visual and audio events are perceptually grouped, facilitating the differentiation of these signals from asynchronous visual distracters. Importantly, both this study and Van der Burg et al. [41], used clearly characterised and temporally transient audio and visual events; two factors that may explain a difference between rapid AV temporal grouping, such as revealed in this study and in the ‘Pip and Pop’ effect [41], [43], and other findings that are more consistent with a serial process for combining audio and visual signals ([35], [44]; see [43] for an investigation of the necessary stimulus conditions for rapid AV temporal grouping).

While our stimuli better approximate cluttered naturalistic conditions than many AV timing experiments, they remain highly abstracted. They are unnatural in that we have presented two or three identical visual events, but only a single auditory event. In our speech stimuli, we have also intentionally made use of a ballistic utterance that we thought might be well suited for precise timing judgments. Therefore, it remains to be seen to what extent our results will generalize. While making this note of caution, we hasten to point out that these issues do not undermine the theoretical significance of our data. We believe that we have shown that the sensitivity of human AV timing perception has been systematically underestimated by experiments that use isolated pairs of audio and visual events, as temporally proximate cross modal events tend to group perceptually, and to seem synchronous as a consequence.

We take our data as evidence that human AV timing perception is shaped by *selective* perceptual grouping processes. While in this study we have demonstrated this using AV pairs, we anticipate that this perceptual strategy will be more generally applicable to combinations of events encoded in other sensory modalities [45]–[47] and to combinations of events encoded within a single sensory modality [48]–[51]. Moreover, we anticipate that the selective perceptual grouping processes we have identified will prove to be important in daily life, as humans need to identify relationships between multisensory events encountered within a complex, cluttered, environment.

## Supporting Information

Movie S1 Example Sequential trial from Experiment 1.
(MOV)

Movie S2 Example Concurrent trial from Experiment 1.
(MOV)
